# Whole-genome surveillance supports hazard profiling of *Escherichia coli* lineages in recycled water treatment systems

**DOI:** 10.1128/aem.01342-26

**Published:** 2026-09-15

**Authors:** Dewa A. P. Rasmika Dewi, Harshanie Abeywardena, Dieter Bulach, Chi-Wen Tseng, Lamiya Bata, Fiona Lynch, Melita Stevens, Nicholas D. Crosbie, Rebekah Henry

**Affiliations:** 1 School of Public Health and Preventive Medicine, Faculty of Medicine, Nursing and Health Sciences, Monash University 2541, Melbourne, Victoria, Australia; 2 Melbourne Water Corporation, Melbourne, Victoria, Australia; 3 Melbourne Veterinary School, Faculty of Science, University of Melbourne 188674 , Parkville, Victoria, Australia; 4 One Water Laboratory, Department of Civil and Environmental Engineering, Monash University 541007, Clayton, Victoria, Australia; University of Minnesota Twin Cities, St. Paul, Minnesota, USA

**Keywords:** recycled water, *Escherichia coli*, whole genome sequencing, antimicrobial resistance, virulence factors, microbial hazard framework, water reuse

## Abstract

The use of treated wastewater is increasingly important for sustainable water management under a changing climate, yet conventional monitoring based on *Escherichia coli* enumeration provides limited insight into strain diversity and associated public health hazards. Here, we applied longitudinal whole-genome sequencing (WGS) to 180 *E. coli* isolates collected across the treatment continuum of a recycled water facility, from influent to final effluent. Genomic analysis revealed extensive strain-level heterogeneity, comprising 88 sequence types across eight phylogroups, with greater diversity in influent than in treated effluent. Phylogenetic comparisons with contextual Australian genomes indicated clustering with strains associated with companion animals, wild birds, humans, and livestock, suggesting multiple potential source reservoirs rather than a single dominant origin, although source contributions were not definitive. Despite a >90% reduction in total *E. coli* loads, isolates recovered from upstream and downstream stages exhibited broadly comparable virulence factor and antimicrobial resistance gene (ARG) profiles, suggesting that, within the cultured isolate collection, reductions in abundance exceeded shifts in genomic composition. To assess operational relevance, we prototyped a genomics-informed hazard framework integrating virulence determinants, ARGs, plasmid-associated mobility, and reuse-specific exposure context. Using this framework, 92.8% of isolates were classified as low hazard, and 7.2% as moderate hazard, with no isolates meeting criteria for high or critical hazard classifications. These findings demonstrate that genomic profiling of indicator organisms can reveal population structure and hazard heterogeneity not captured by conventional enumeration alone, and can provide a practical basis for incorporating genomic information into hazard-informed monitoring of recycled water systems.

**IMPORTANCE:**

Routine recycled water monitoring relies largely on culture-based *E. coli* counts, which indicate regulatory compliance but provide limited insight into strain diversity, persistence, and genomic characteristics relevant to public health. Using longitudinal whole-genome sequencing, we show that genetically distinct *E. coli l*ineages, including isolates carrying combinations of virulence and antimicrobial resistance determinants, can persist through advanced treatment despite substantial reductions in overall *E. coli* loads. While most isolates were classified as low genomic hazard and no high- or critical-hazard isolates were detected, these findings demonstrate that conventional enumeration alone cannot distinguish between genetically diverse lineages with differing hazard potential in highly treated systems. By integrating genomic data into a hazard classification framework, this study demonstrates an applied approach to contextualize *E. coli* detections and distinguish low-risk background populations from isolates with elevated genomic hazard profiles. This work supports the use of genomic profiling of indicator organisms to improve surveillance, inform treatment performance assessment, and enable more risk-based management of recycled water systems.

## INTRODUCTION

Increasing global water demand, driven by climate variability and population growth, has accelerated the adoption of wastewater recycling as a key component of sustainable water management (1). Recycled water is now widely used in agricultural irrigation, industry, environmental augmentation, and, in some regions, indirect or direct potable reuse (2). As reliance on water reuse increases, ensuring microbial safety across diverse end uses remains a critical operational and regulatory challenge.

*Escherichia coli* is widely used as a regulatory indicator of fecal contamination and treatment performance. However, this species exhibits substantial genomic diversity, encompassing environmentally persistent commensals as well as lineages carrying virulence factors (VFs) and antimicrobial resistance genes (ARGs) of clinical significance (3, 4). Consequently, equivalent culture-based counts may represent markedly different public health risks. Although ultraviolet (UV) irradiation and chlorination effectively reduce microbial loads (5), viable *E. coli* can still be detected following treatment. Evidence from environmental and engineered systems indicates that some strains can persist in water, sediments, or soils and tolerate fluctuating environmental conditions (6–8). In recycled water schemes, such persistence raises important questions regarding lineage-specific resilience and the distribution of clinically relevant genetic traits.

Whole-genome sequencing (WGS) enables high-resolution characterization of *E. coli* lineage structure, accessory genome diversity, and the presence of virulence determinants, ARGs, and mobile genetic elements (MGEs) (9, 10). While genomic approaches have been applied to natural aquatic environments (11, 12), their integration into routine assessment of recycled water systems remains limited (13). Engineered treatment systems provide a controlled context in which inputs, processes, and operational conditions are defined, enabling evaluation of how treatment influences microbial population structure. Genomic analysis in these systems supports surveillance beyond binary compliance metrics and enables more resolved assessment of microbial hazard.

Integrating genomic data with operational metadata, including treatment stage, temporal variation, and end-use exposure context, can improve hazard assessment and support evidence-based decision-making. Identification of persistent lineages and clinically relevant traits provides actionable information for optimizing treatment performance and managing risk across reuse applications.

This study applied longitudinal whole-genome sequencing to characterize lineage diversity, persistence across treatment stages, and the distribution of VFs, ARGs, and plasmids in an engineered recycled water system. These data were integrated with system-level and end-use information to evaluate a genomics-informed hazard framework for surveillance and decision-making.

## RESULTS

### Spatial-temporal dynamics and environmental drivers of *E. coli* loads

To examine spatial variation within the recycled water system, *E. coli* loads (MPN/100 mL) were compared across sampling sites and seasons. Significant spatial variation was observed, with the highest *E. coli* concentrations in influent samples and substantially lower concentrations following treatment. Water samples from W1 exhibited the highest loads, with a median log₁₀(MPN + 1) of 1.82 (IQR: 1.57–2.07), corresponding to 64.5 MPN/100 mL. In comparison, W3 had a median of 0.30 (IQR: 0.18–0.64), and W2 showed the lowest levels (median = 0, IQR: 0–0.18) (Table S1A). Wilcoxon rank-sum tests confirmed significant differences between all sources: W1 vs W2 (W = 1119, *P* = 8.18 × 10⁻¹³), W1 vs W3 (W = 2869, *P* < 2.2 × 10⁻¹⁶), and W3 vs W2 (W = 520, *P* = 1.45 × 10⁻⁷) (Fig. S1A).

Temporal patterns were also examined, and correlation between overall *E. coli* load and physicochemical parameters (pH and temperature) were assessed. *E. coli* loads were higher in winter (median = 1.91; IQR: 0.35–2.14) than in other seasons (spring: 0.35; summer: 0.48; autumn: 0.48). However, the overall season effect was not significant (*χ²* = 6.20, *df* = 3, *P* = 0.102) (Table S1B, Fig. S1B). Dunn’s post-hoc tests showed no significant pairwise differences. The spring–winter comparison approached significance (*P* = 0.091), whereas all other comparisons were not significant (Table S1C).

To explore environmental drivers, *E. coli* concentration data (log₁₀(MPN + 1)) were correlated with physicochemical parameters at W1 and W3. No physicochemical data were available for W2; therefore, these samples were excluded. Aggregated data from W1 and W3 showed a significant positive correlation with pH (Spearman’s *ρ* = 0.41, *P* < 0.001; Fig. S1C) and a weak negative correlation with temperature (*ρ* = –0.26, *P* = 0.040; Fig. S1D). However, these associations were not observed when analyzed by site (pH: W1 *ρ* = –0.31, *P* = 0.092; W3 *ρ* = –0.12, *P* = 0.520; temperature: W1 *P* = 0.158; W3 *P* = 0.687). For dissolved oxygen (DO), no association was observed in aggregated data (*ρ* = –0.10, *P* = 0.456; Fig. S1E), whereas a moderate negative correlation was detected at W1 (*ρ* = –0.41, *P* = 0.024).

### Genomic diversity of *E. coli* isolates and potential source attribution

All 180 genomes passed quality control thresholds (>90% completeness and <5% contamination). Sequencing coverage ranged from 21× to 393× (median = 45× ). Sequencing coverage ranged from 21× to 393× (median = 45×). Assemblies comprised 1–25 contigs per genome, with N50 values ranging from 0.28 to 5.22 Mb (median = 1.46 Mb) (Table S2). These assembly metrics indicate high-quality draft genomes suitable for downstream comparative genomic and phylogenetic analyses. Whole-genome sequencing of 180 *E. coli* isolates (W1: *n* = 85; W2: *n* = 11; W3: *n* = 84) revealed high genomic heterogeneity, encompassing 88 distinct sequence types (STs). The most common STs were ST155 and ST162 (each *n* = 8; 4.4%) (Table S2). Twenty-five isolates (13.9%) possessed allelic profiles that did not match existing sequence types in EnteroBase and were therefore classified as untyped STs. Requests for assignment of new ST designations have been submitted and are currently under review by the EnteroBase curators.

Isolates from W1 exhibited greater ST richness (56 unique STs) compared to those from W3 (42 unique STs). Phylogroup distribution spanned eight groups, with B1 predominating (*n* = 120 isolates, 66.7%), followed by A (*n* = 23, 12.8%), B2 (*n* = 11, 6.1%), D (*n* = 11, 6.1%), and E (*n* = 9, 5%). Less common phylogroups included C (*n* = 3; 1.7%), G (*n* = 1; 0.6%), and H (*n* = 1; 0.6%), and one isolate was unassigned (unknown, *n* = 1; 0.6%) (Table S2).

The total gene content per genome ranged from 4,073 to 5,067 genes (Table S3). Mean gene counts were similar between W1 (4,440 ± 136) and W3 (4,521 ± 191), with comparable ranges across sites (Table S3, Fig. S2). Due to the limited number of isolates recovered from W2 (*n* = 11) and their restricted temporal coverage (October 2024–January 2025 and May 2025), W2 was excluded from formal statistical comparisons to avoid bias associated with unequal sample sizes and sampling effort. Gene richness differed significantly between W1 and W3 (Kruskal–Wallis *χ*² = 6.53, df = 1, *P* = 0.011), although the absolute difference was modest. PERMANOVA based on Jaccard distances indicated a small but significant shift in gene composition between sites (*R*² = 0.016, *F*_₁,₁₆₇_ = 2.65, *P* = 0.002). Homogeneity of dispersion was not significant (*F*_₁,₁₆₇_ = 2.01, *P* = 0.159).

**Fig 1:**
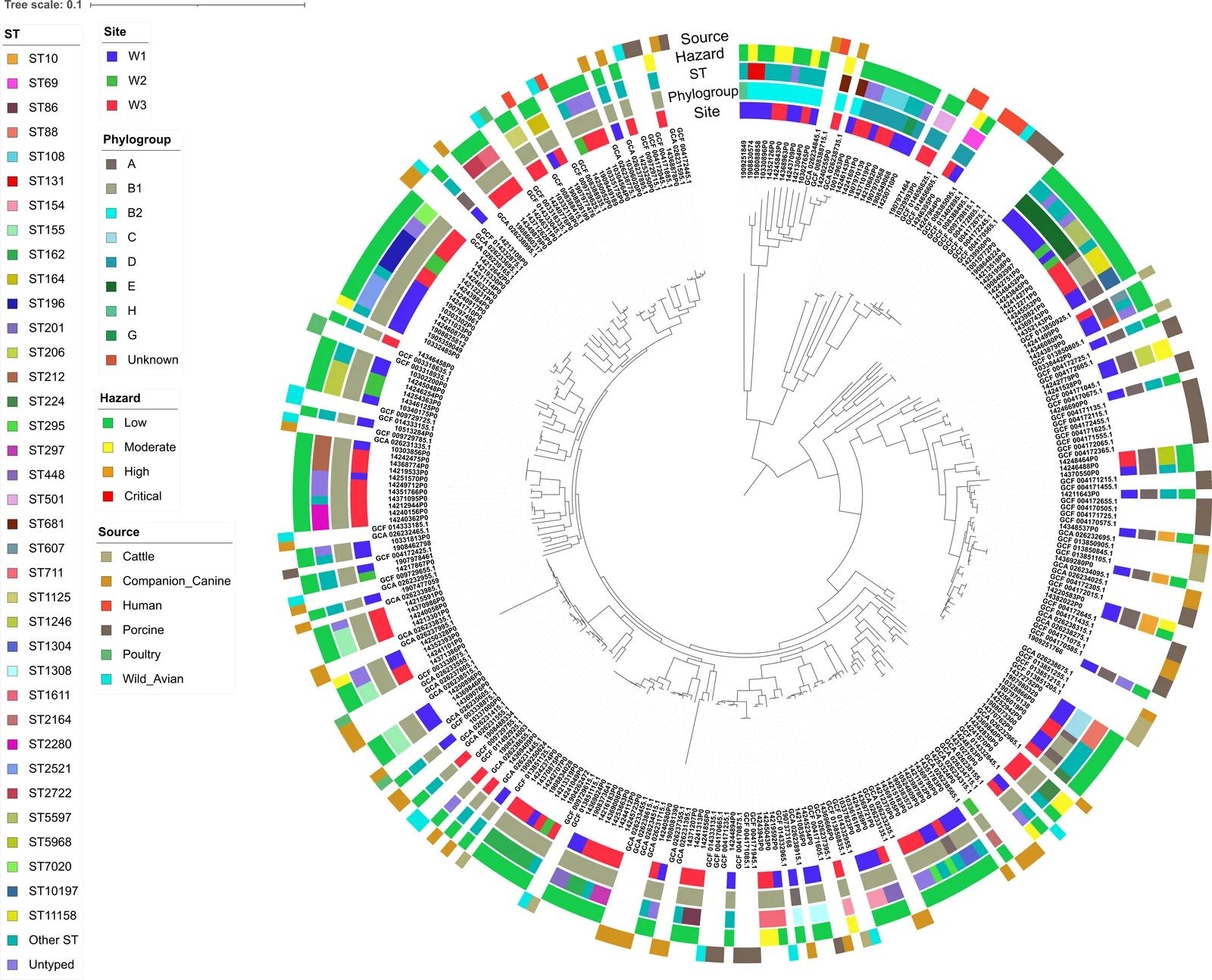
Core genome phylogenetic tree of 180 *E. coli* isolates from the recycled water system and 121 contextual *E. coli* genomes from historical Australian collections sharing ≥99% average nucleotide identity (ANI). The tree was constructed from a core-genome SNP alignment using IQ-TREE v2.1.3 with 1,000 bootstrap replicates and visualized using iTOL (https://itol.embl.de/). Metadata tracks adjacent to each tip indicate sampling location, phylogroup, sequence type (ST), genomic hazard classification, and the associated isolation source of the Australian reference genomes (companion animal, livestock, wild animal, human, and wastewater). For visualization purposes, STs represented by more than two isolates were assigned individual colors in the ST legend, whereas STs detected in two or fewer isolates were grouped and displayed as “Other ST.” Untyped isolates were displayed separately as “Untyped.” The tree was midpoint rooted.

Phylogenetic analysis of the 180 study isolates together with 121 closely related Australian reference genomes (ANI ≥99% to at least one study isolate) is shown in Fig. 1. The majority of isolates (*n* = 68, 37.8%) clustered most closely with companion animal-associated contextual genomes, primarily canines, followed by wild birds (*n* = 44, 24.4%), humans (*n* = 25, 13.9%), and livestock (pigs: 23 isolates, 12.8%; cattle: six isolates, 3.3%) (Table S4, Fig. S3A). Smaller proportions were similar to poultry-associated strains (*n* = 12, 6.7%), wastewater (*n* = 1, 0.6%), and feline sources (*n* = 1, 0.6%). Source distributions showed evidence of variation between W1 and W3 (Fisher’s Exact test, *P* = 0.027), although these patterns should be interpreted cautiously given the sampling design and isolate numbers. While companion animal associations were similar at both sites (~40%), secondary associations differed, with W1 more frequently linked to porcine sources and W3 to wild birds (Fig. S3B). These findings suggest that *E. coli* populations within the recycled water system likely originate from multiple host reservoirs rather than a single dominant source. The increased representation of wild bird-associated lineages among W3 isolates may reflect persistence through treatment, ecological selection within the post-treatment environment, residence within the contact basin or associated infrastructure, or other post-treatment environmental processes.

### Composition and population structure of *E. coli* isolates

**Fig 2:**
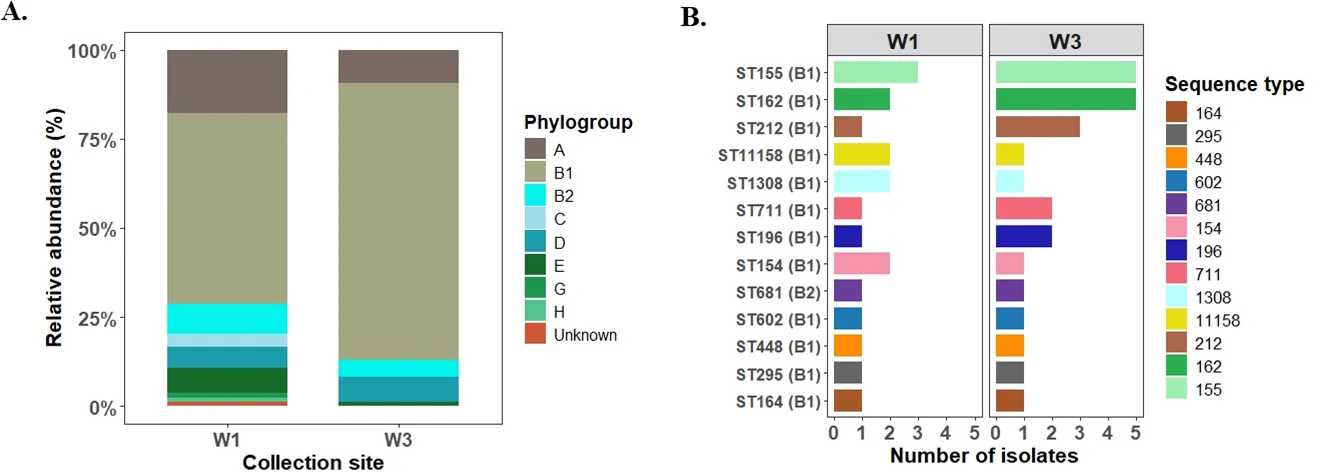
(**A**) Proportional distribution of *E. coli* phylogroups in influent (W1) and post-treatment (W3) samples. (**B**) Distribution of the 10 most prevalent sequence types detected in both influent (W1) and post-treatment (W3) samples, excluding untyped isolates (NT). Sequence types are ordered by overall frequency, and their corresponding phylogroups are indicated alongside each ST label.

Phylogroup compositions differed among sites (Fisher’s exact test, *P* = 0.010), with rare phylogroups (C, G, and H; *n* < 5) grouped as “Other.” Although statistically significant, these results should be interpreted cautiously because unequal isolate recovery across sampling locations may have influenced the observed distributions. Both W1 and W3 were dominated by phylogroup B1, but W1 showed greater heterogeneity, whereas W3 lacked several minor phylogroups (Fig. 2A). Correspondingly, α-diversity was higher at W1 than W3 (Shannon: 1.37 vs 0.80; Simpson: 0.65 vs 0.38).

Sequence type distributions also suggested differences across sites (Fisher’s exact test, *P* = 2 × 10^−4^; W1 vs W3, *P* = 0.003). Diversity indices indicated slightly higher richness and evenness at W1 (Shannon 3.85; Simpson 0.975) than at W3 (3.66; 0.970). Several STs were shared between sites, including ST162, ST155, and ST212 (Fig. 2B), suggesting the possibility of persistence or repeated introduction of certain lineages across treatment stages. However, confirmation of these patterns would benefit from a larger sample size to improve statistical confidence. SNP analysis of the most prevalent STs (ST162: *n* = 7 and ST155: *n* = 8) revealed contrasting patterns. ST162 included near-identical genomes (≤1 SNP) across sites and timepoints, as well as highly divergent pairs (>3,000 SNPs), indicating co-occurrence of closely related and distinct lineages (Table S5A). In contrast, ST155 isolates showed substantial divergence, with only two closely related intra-site pairs (<10 SNPs) and no closely related inter-site pairs (Table S5B). These findings suggested that ST155 populations were largely composed of highly diverse lineages with only limited evidence of local clonal expansion.

### Virulence determinants and antibiotic resistance profiles

**Fig 3:**
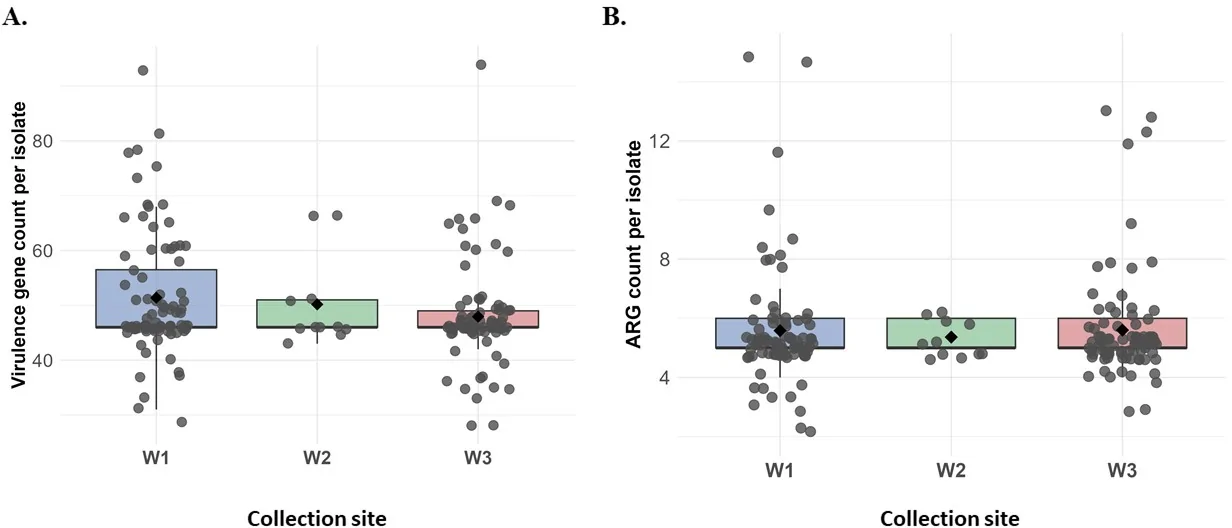
Richness of virulence factors (VFs) and antibiotic resistance genes (ARGs) in *Escherichia coli* isolates across collection sites (W1, W2, W3). (**A**) Boxplots show the distribution of VF counts per isolate at each site. Boxes represent the interquartile range, the horizontal line indicates the median, and gray dots show individual isolates. The black diamond marks the mean VF count for each site. Colors correspond to collection sites. (**B**) Boxplots of the total number of ARG counts per isolate at each site, irrespective of whether individual resistance determinants represent intrinsic resistance mechanisms in *E. coli*. The same visual conventions as in panel **A** are used: interquartile range (boxes), median (line), individual isolates (gray dots), and mean (black diamond).

A total of 195 distinct virulence genes were detected across all isolates (Table S6). The number of unique genes detected per site differed (W1: 181; W3: 127; W2: 68), although this likely reflects differences in sample size. The median number of virulence genes per genome was identical across sites (median = 46), with similar variability (Fig. 3A). No significant difference was observed between W1 and W3 (W = 4114, *P* = 0.082). Presence–absence analysis of virulence gene profiles revealed only minor differences between the W1 and W3 isolates. Although PERMANOVA detected a statistically significant effect of collection site on virulence composition (*R*² = 0.013, *P* = 0.041), site explained only 1.3% of the total variation, and dispersion analysis indicated unequal within-site variability (betadisper *P* = 0.024), suggesting broadly conserved virulence repertoires across treatment stages.

A total of 47 distinct ARGs were identified across the isolate collection (Table S6). Site-level ARG richness was highest in W1 (39 ARGs) and W3 (34 ARGs) and lowest in W2 (seven ARGs). Median ARG counts per genome were similar across sites (~5 genes per genome), with no significant difference between W1 and W3 (Wilcoxon rank-sum test: W = 3596, *P* = 0.928) (Fig. 3B). The dominant resistance-associated profiles were broadly similar between influent and post-treatment isolates. Efflux-associated genes and β-lactam determinants were detected in almost all isolates from both W1 and W3, while fosfomycin-associated genes occurred in more than 90% of isolates at both sites (Fig. S4). Colistin-associated determinants were more prevalent in W3 but were almost entirely represented by chromosomal *pmrB* variants (*pmrB_Y358N* and *pmrB_E123D*) rather than acquired *mcr* genes. Other resistance subclasses, including quinolone, sulfonamide, tetracycline, and cephalosporin determinants, occurred at low frequencies and exhibited similar prevalence across treatment stages (Fig. S4). Overall, W1 and W3 exhibited broadly similar resistance profiles despite differences in isolate abundance across sites.

### Genomic hazard assessment framework

**Fig 4:**
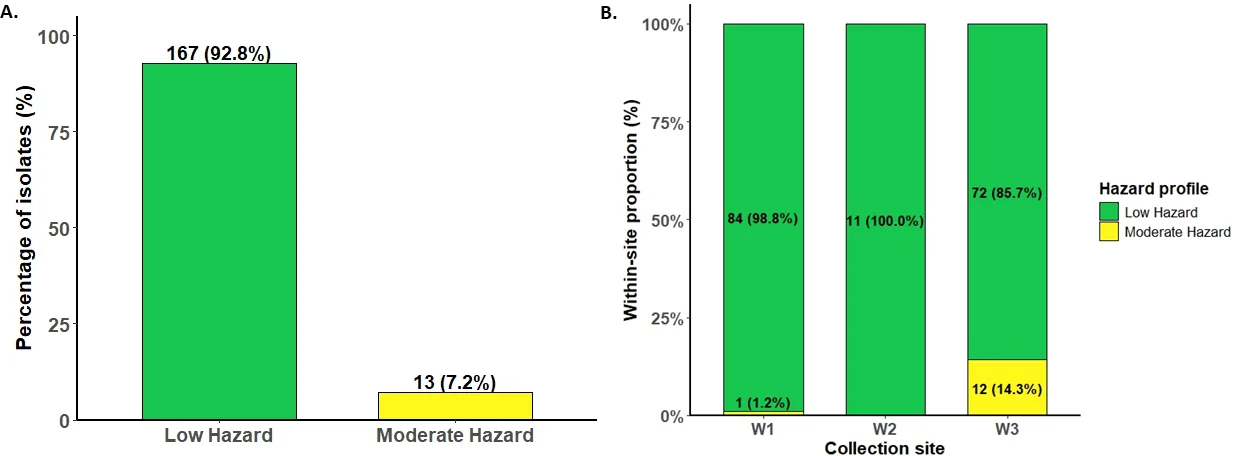
Genomic hazard profile of *E. coli* isolates recovered from the recycled water system. (**A**) Overall hazard distribution of isolates classified as low (green), moderate (yellow), high (orange), or critical (red) according to the genomic hazard assessment framework. Bars represent the percentage of isolates assigned to each hazard category, with labels indicating the number and proportion of isolates (*n*, %). (**B**) Hazard profile across sampling sites (W1, W2, and W3). Stacked bars show the within-site proportion of isolates assigned to each hazard category, with labels indicating both the number of isolates and the corresponding percentage within each site (*n,* %).

The genomic hazard assessment framework integrated virulence determinants, antimicrobial resistance genes, and plasmid-associated mobility into a composite hazard classification interpreted within the context of recycled water end use (EAHI) (Table S7). Overall, most isolates were classified as low hazard (92.8%, *n* = 167), while a smaller proportion were classified as moderate hazard (7.2%, *n* = 13) (Table S7; Fig. 4A). No isolates met the criteria for high or critical hazard classifications.

Hazard profiles varied across sampling locations (Fig. 4B). Influent samples (W1) were predominantly composed of low-hazard isolates (98.8%, *n* = 84), with one isolate classified as moderate hazard (1.2%). All isolates recovered from the intermediate treatment stage (W2) were classified as low hazard (*n* = 11). Post-treatment samples (W3) contained a higher proportion of moderate-hazard isolates (14.3%, *n* = 12), although low-hazard isolates remained predominant (85.7%, *n* = 72). The hazard-profile distribution differed significantly between W1 and W3, with lower odds of moderate versus low hazard at W1 than at W3 (Fisher’s exact test, *P* = 0.0012; OR = 0.07, 95% CI = 0.002–0.51).

## DISCUSSION

Routine microbial monitoring of recycled water has traditionally relied on culture-based enumeration of *E. coli* to assess treatment performance and regulatory compliance (14, 15). While cost-effective and operationally useful, this approach provides limited resolution of strain diversity and ecological behavior. In contrast, genomic profiling provides higher-resolution insight into population structure, persistence, and the distribution of virulence and resistance determinants in engineered water systems. This improved resolution enables differentiation between low-risk background detections and isolates carrying genomic features associated with elevated hazard potential (9, 10).

### Dynamics of *E. coli* load and environmental drivers

Spatial patterns confirmed substantial reduction in *E. coli* loads along the treatment train, with highest concentrations at W1 and lowest at downstream sites. Although winter medians were higher, seasonal differences were not statistically significant, suggesting that short-term variability rather than consistent seasonal effects likely explains observed fluctuations (16, 17).

Correlations between *E. coli* loads and physicochemical parameters were observed in aggregated data but not within individual sites. This indicates that these relationships likely reflect differences between treatment stages rather than within-site environmental effects. A moderate negative association between dissolved oxygen and *E. coli* at W1 may reflect localized ecological conditions; however, effect sizes were modest and should be interpreted cautiously. Overall, these findings suggest that treatment processes are the primary determinants of *E. coli* abundance, with physicochemical variation playing a secondary role within the observed operational range.

### Genomic diversity and source attribution

Whole-genome sequencing revealed substantial genomic heterogeneity, encompassing 88 sequence types across multiple phylogroups. The predominance of B1 and A lineages is consistent with environmentally adapted populations commonly observed in aquatic systems (18–20), whereas the presence of B2 and D phylogroups indicates occasional occurrence of lineages associated with higher virulence potential (21, 22). Reduced phylogroup and ST diversity at W3 relative to W1 suggests selective filtering along the treatment continuum, with downstream dominance of environmentally resilient lineages.

Phylogenetic comparisons indicated similarity to strains associated with companion animals, wild birds, humans, and livestock, suggesting that the *E. coli* population within the recycled water system likely reflects multiple fecal reservoirs rather than a single dominant source. Companion animal-associated lineages were the most frequently identified, consistent with increasing recognition of pets as reservoirs of environmentally disseminated *E. coli*, while the substantial contribution of wild bird-associated lineages may reflect interactions between recycled water infrastructure and surrounding wildlife. Differences in inferred source distributions between W1 and W3 may reflect treatment-associated selection, differences in environmental exposure following treatment, or limitations inherent to reference-based source attribution approaches.

Accumulation of organic material may support localized persistence or regrowth of *E. coli* (23, 24). Importantly, source attribution based on ≥99% ANI should be interpreted cautiously, as similarity to reference genomes reflects phylogenetic relatedness and ecological association rather than definitive host origin, particularly for generalist B1 lineages (25, 26). Nevertheless, these findings highlight the utility of whole-genome sequencing for providing source-related context that is not obtainable through conventional *E. coli* enumeration. Such information may assist water utilities in identifying likely contamination pathways, evaluating treatment performance, and prioritizing management interventions aimed at reducing microbial hazards within recycled water systems.

Although differences in gene composition between W1 and W3 were statistically significant, the effect size was small (PERMANOVA *R*² = 1.6%). Therefore, these findings should be interpreted as modest shifts in population structure rather than major changes in the overall genomic composition of *E. coli* across the treatment continuum. Further studies incorporating larger and more balanced isolate collections would help clarify the extent to which these patterns are representative of *E. coli* populations within recycled water systems.

### Lineage dynamics and persistence

SNP analysis revealed lineage-specific dynamics within dominant sequence types. ST162 included both near-identical genomes across sites and highly divergent lineages, indicating co-occurrence of closely related and distinct strains within the same MLST group. The presence of near-identical isolates across sites and timepoints is consistent with short-term persistence or repeated introduction, whereas highly divergent pairs likely reflect independent inputs or longer-term diversification.

In contrast, ST155 isolates showed substantial genomic divergence, with limited evidence of close relatedness and no closely related inter-site pairs. This pattern is consistent with repeated introduction rather than sustained persistence within the treatment system. These findings highlight variability in lineage behavior and demonstrate the value of genome-scale analysis for distinguishing persistence from reintroduction in engineered water systems (23, 27).

### Virulence and antimicrobial resistance profiles

Across all isolates, 195 virulence genes and 47 ARGs were detected. Despite differences in site-level gene richness, the number of virulence genes per genome was consistent across sites, indicating that treatment reduced overall diversity without substantially altering per-genome virulence content. Many detected virulence determinants were associated with adhesion, iron acquisition, secretion systems, and colonization functions that may contribute to environmental persistence and host interaction (28, 29). This pattern is consistent with selection for traits that enhance survival under nutrient-limited and intermittently disinfected conditions (30–32).

ARG abundance per genome did not differ significantly between sites, suggesting that treatment influenced lineage composition without substantially changing the resistance gene burden of surviving populations. Efflux-associated genes were ubiquitous across isolates; however, their prevalence should be interpreted cautiously because many represent intrinsic components of the *E. coli* genome that contribute to baseline resistance phenotypes rather than acquired antimicrobial resistance or elevated clinical risk (**33**).

### Genomic hazard framework and implications

Integration of virulence, resistance, and plasmid mobility determinants into a composite framework indicated that most isolates recovered from the recycled water system were classified as low hazard, with a small proportion classified as moderate hazard. Moderate-hazard isolates were more frequent in post-treatment samples (W3) than in influent samples (W1), and the hazard-profile distribution differed significantly between these sites (Fisher’s exact test, *P* = 0.0012). Although low-hazard isolates remained predominant at all sampling locations, the higher proportion of moderate-hazard isolates at W3 indicates that treatment-stage differences in the composition of the recovered *E. coli* population may warrant further consideration.

The predominance of low-hazard isolates is consistent with the generally low pathogenic potential expected among environmental *E. coli* populations and supports the interpretation that the *E. coli* population recovered from the recycled water system was predominantly composed of isolates with relatively low genomic hazard potential. Nevertheless, the detection of moderate-hazard isolates following treatment highlights the value of genomic surveillance approaches capable of identifying isolates with elevated virulence, resistance, or plasmid mobility characteristics that may not be distinguished by conventional indicator enumeration alone. These findings demonstrate that genomic approaches can provide additional resolution beyond traditional culture-based monitoring by identifying less abundant isolates with potentially greater public health relevance. Integration of such frameworks into monitoring programs may therefore improve interpretation of *E. coli* detections and support more targeted hazard assessment and management (34, 35).

### Limitations and future directions

Several limitations should be considered. First, this study was conducted at a single facility, and observed patterns may reflect site-specific conditions. Broader validation across systems and treatment configurations is needed to assess generalizability. Second, although W3 represents the most downstream stage of the treatment train and the final recycled water product, samples were collected from an effluent tap downstream of a closed contact basin following completion of all treatment processes. Consequently, the presence of isolates exhibiting genomic similarity to companion animal-, livestock-, or wildlife-associated contextual genomes cannot be attributed solely to treatment performance and may reflect a combination of persistence through treatment, prolonged hydraulic residence within the contact basin, or survival within downstream infrastructure such as biofilms. Distinguishing among these mechanisms was beyond the scope of the present study and would require targeted environmental sampling together with higher-resolution source-tracking approaches.

Third, the number of *E. coli* isolates recovered differed substantially among sampling locations, reflecting differences in *E. coli* abundance across treatment stages. In particular, only a limited number of isolates were recovered from W2 following advanced treatment and disinfection, restricting statistical comparisons involving this site. Although appropriate non-parametric statistical approaches were used and W2 was excluded from formal comparative analyses, the unequal sampling structure may have reduced statistical power and limited the representativeness of some comparisons. Consequently, observed differences in genomic diversity, source attribution, and hazard profiles should be interpreted as indicative trends within the sampled isolate collection rather than definitive population-level estimates.

Fourth, the isolate-based approach does not capture the full microbial community, including uncultivable organisms or low-abundance populations. Future studies incorporating shotgun metagenomics would provide a more comprehensive assessment of microbial diversity, antimicrobial resistance determinants, virulence-associated genes, and potential pathogen reservoirs within recycled water systems. Finally, the proposed framework represents a prototype approach and has not yet been validated against epidemiological outcomes, environmental surveillance data, or quantitative microbial risk assessment (QMRA) models. Future studies incorporating larger isolate collections, longitudinal sampling, phenotypic validation, and integration with exposure modeling will be important for refining and validating genomic hazard assessment approaches for water reuse applications.

Despite these limitations, the study highlights the potential value of integrating whole-genome sequencing into routine recycled water surveillance programs and provides a foundation for the development of a validated hazard-informed hazard assessment framework for water reuse systems.

Overall, genomic analysis of *E. coli* in a recycled water system revealed high lineage diversity, limited but detectable site-level structuring, and predominantly low-hazard populations, with a small proportion of moderate-hazard isolates detected across treatment stages. These findings indicate that genome-resolved analysis improves interpretation of *E. coli* detections beyond conventional enumeration and provides a practical basis for incorporating genomic data into hazard-informed monitoring of recycled water systems.

## MATERIALS AND METHODS

### Sample and strain collection

The study was conducted at a recycled water plant in Victoria, Australia, a 10,500-ha lagoon-based wastewater treatment and recycling facility that processes approximately 600 ML per day of predominantly domestic municipal wastewater, with additional urban and industrial trade waste inputs. The facility produces approximately 30,000 ML of recycled water annually for environmental and agricultural reuse. Although the influent does not receive direct agricultural runoff or concentrated livestock effluent streams, the site is situated within a mixed agricultural and wildlife landscape, with extensive livestock grazing areas and internationally significant wetlands supporting large populations of migratory and resident waterbirds. Consequently, opportunities for secondary environmental fecal deposition within open lagoon systems cannot be excluded.

**Fig 5:**
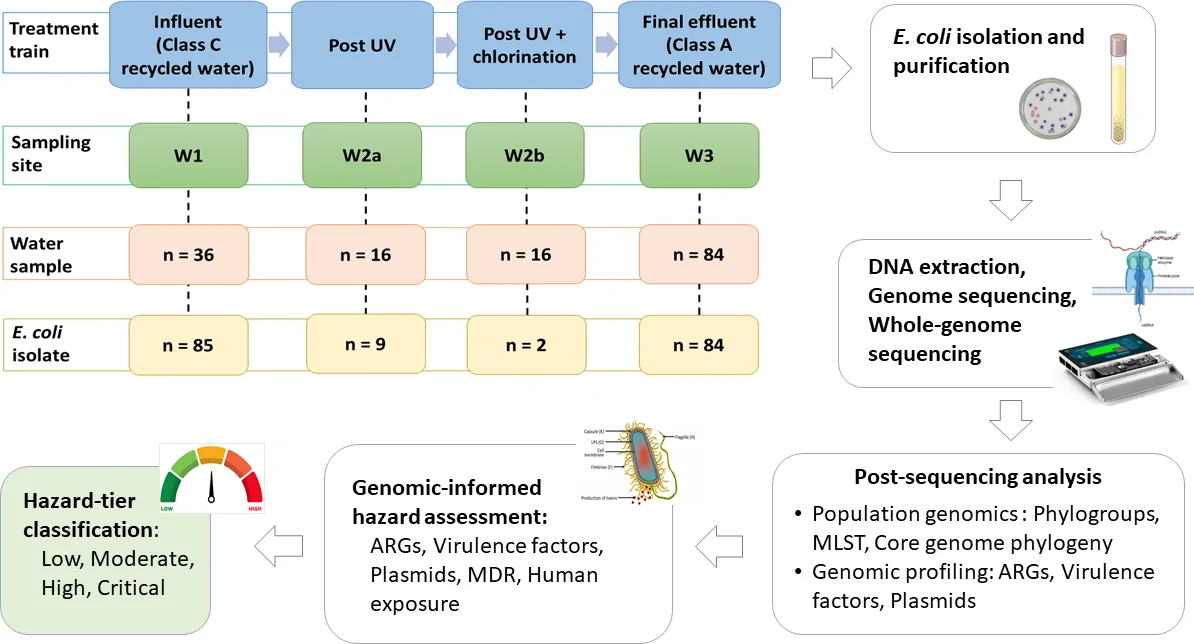
Overview of the study design and analytical workflow. Water samples were collected from influent Class C recycled water (W1), post-UV treatment (W2a), post-UV plus chlorination treatment (W2b), and final Class A recycled water stored in the contact basin prior to distribution (W3). Sampling yielded 180 *E. coli* isolates, which were subjected to culture purification, Oxford Nanopore whole-genome sequencing, genome assembly, taxonomic validation, phylogenetic and population genomic analyses, and characterization of virulence factors, antimicrobial resistance genes (ARGs), and plasmid-associated mobile genetic elements. Results were integrated into a genomic hazard framework incorporating virulence potential, ARG hazard, multidrug resistance breadth, plasmid hazard, and potential human exposure pathways.

Water samples (*n* = 154) were collected as grab samples between January 2024 and June 2025 from three locations within a recycled water treatment system: (i) treated wastewater influent entering the recycled water treatment system (W1, *n* = 36), (ii) post-treatment water following UV disinfection (W2a, *n* = 16) or UV disinfection followed by chlorination (W2b, *n* = 16), and (iii) final recycled water stored within the contact basin prior to distribution for Class A recycled water reuse applications (W3, *n* = 84) (Fig. 5; Table S8). In addition, two sludge-associated samples were collected from the W3 contact basin during routine maintenance activities (W3 sludge, *n* = 2).

Samples were processed following standard filtration procedures outlined in AS/NZS 4276.5:2019 at the One Water Laboratory (Monash University, Victoria, Australia). For *E. coli* isolation, 100 mL aliquots were filtered in duplicate through 0.45 µm membrane filters and cultured on MI agar (Edwards Group, Australia) at 37°C for 18–24 h. In parallel, positive Colilert trays obtained from routine *E. coli* quantification at W1, W2, and W3 by Australian Laboratory Services Global (Scoresby, VIC, Australia) were used as an additional source of *E. coli* isolates. Up to three presumptive *E. coli* colonies per sample were randomly selected from MI agar, subcultured onto MacConkey and XLD agar (Oxoid; BD Difco), and purified through successive streaking prior to downstream genomic analysis. All isolates were archived in the One Water Biobank under ISO9001-certified procedures.

### DNA extraction and sequencing

Single colonies were cultured in LB broth and incubated at 37°C for 18–24 h, and genomic DNA was extracted using the Quick-DNA Miniprep Kit (Zymo Research, USA) according to the manufacturer’s instructions. DNA was quantified using a Qubit fluorometer (Invitrogen), and ≥200 ng was used for library preparation.

Sequencing libraries were prepared using the Oxford Nanopore Rapid Barcoding Kit (SQK-RBK114, Oxford Nanopore, UK). Pooled libraries were purified and loaded onto an R10.4.1 MinION flow cell (FLO-MIN114) for 48 h at the Monash Genomics and Bioinformatics Platform. Base-calling of FAST5 files was performed using Dorado v0.5.1 in super-accuracy (SUP) mode.

### Genome assembly and quality assessment

Raw read statistics were summarized using Seqkit v2.10.0 (36). *De novo* assembly for long-reads was performed with Dragonflye v1.2.1 (https://github.com/rpetit3/dragonflye) using default parameters, which included adaptor trimming, removal of reads <1,000 bp, assembly with Flye, polishing with Racon, and filtering short, low-coverage, or homopolymer-rich contigs. Final polishing was completed with Medaka v2.0.1 (https://github.com/nanoporetech/medaka) using the r1041_e82_400bps_sup_v5.0.0 model.

A minimum sequencing depth of >20× per genome was targeted. Recent benchmarking of bacterial SNP calling using Oxford Nanopore R10.4.1 data with super-accuracy (SUP) base calling has shown that accurate SNP inference can be achieved at ~10× coverage, with performance comparable to Illumina-based approaches (37). Accordingly, sequencing depths in this study exceeded conservative thresholds reported to be sufficient for robust SNP-based analyses.

Assembly quality and completeness were assessed using CheckM v1.2.2 (≥90% completeness, <5% contamination) (38) and BUSCO v5.1.3 (Enterobacterales data set, ≥90% completeness) (39). Assembly statistics, including genome size, number of contigs, sequencing depth, GC content, and N50, were calculated using Seqkit v2.10.0 and are provided in Table S2. Taxonomic classification was performed with Kraken2 v2.1.3 (40) against a GTDB-derived database (downloaded May 2024). All analyses were conducted on the M3 High Performing Computing facility (Monash University, VIC, Australia) (https://research.monash.edu/en/equipments/high-performance-computing-m3massive).

### Population genomics and phylogenetic analysis

*In silico* phylogroup assignment was performed using the Clermont Typing (http://clermontyping.iame-research.center/), and sequence types (STs) were determined by multi-locus sequence typing (MLST) v2.15 (Achtman scheme; https://github.com/tseemann/mlst). A contextual data set of 936 Australian *E. coli* genomes (clinical human *n* = 168, livestock_animal *n* = 239, wild_animal *n* = 107, companion animal *n* = 382, wastewater *n* = 40; Table S9) was retrieved from NCBI. Genomes were filtered using CheckM v1.2.2 (≥90% completeness, <5% contamination) (38), and taxonomic identity was confirmed using Kraken2 v2.1.3 (40). Pairwise genomic similarity was assessed using Mash v1.4.6 using default settings (41) and FastANI v1.33 (42). Of the 936 contextual genomes, 121 shared ≥99% ANI with at least one study isolate and were retained for comparative phylogenetic analysis.

Core genome alignments were generated with Roary v3.13.0 (43) using Prokka v1.14.6 annotation (.gff files) (44). Maximum-likelihood phylogenies were inferred using IQ-TREE v2.1.3 with 1,000 ultrafast bootstrap replicates (45). Phylogenetic trees were midpoint rooted for visualization purposes and displayed using iTOL v6 (46) alongside metadata (source attribution, collection date, ST, and phylogroup). For Australian reference genomes, source categories (human, companion animal, livestock, wild animal, and wastewater) were included as metadata tracks to facilitate interpretation of source attribution patterns and relationships with study isolates. SNP distances and clonal relationships were evaluated using SNP-sites v2.5.1 (47) to extract core SNP alignments and SNP-dists v0.6.3 (https://github.com/tseemann/snp-dists) to generate pairwise SNP distance matrices.

### Development of genomic hazard framework

A genomic-based hazard assessment framework was developed to estimate the potential public health relevance of environmental *E. coli* isolates. The framework integrates four genomic dimensions: (i) virulence determinants, representing pathogenic potential; (ii) ARGs, representing the clinical significance of resistance; (iii) multidrug resistance (MDR) breadth, representing resistance diversity across antimicrobial classes; and (iv) plasmid-associated hazard, representing the potential for horizontal dissemination of resistance and virulence determinants. These components were analyzed as separate axes to preserve biological interpretability and were not initially collapsed into a single primary “hazard score.” The framework is intended as a relative genomic screening tool for comparing isolates across treatment stages and exposure contexts.

#### Virulence factor identification and scoring

Virulence-associated genes were identified using ABRicate v1.0.1 (https://github.com/tseemann/abricate), against the VFDB database (48) (≥90% identity and ≥80% coverage). Genes were classified according to established *E. coli* pathotypes, including diarrheagenic *E. coli* (DEC) and extraintestinal pathogenic *E. coli* (ExPEC), including uropathogenic *E. coli* (UPEC).

Key DEC markers included genes associated with enteropathogenic/EPEC (*eae*, *bfpA),* Shiga toxin-producing*/*STEC and hemolytic uremic syndrome/HUS (*stx1*, *stx2*, *ehxA),* enteroaggregative/EAEC (*aggR*, *aaiC*, *aatA)*, enterotoxigenic/ETEC (*eltA/B*, *estA/B)*, enteroinvasive/EIEC, and diffusely adherent/DAEC (*ipaH*, *virF*, *afa*, *daaE*) (28, 29, 49).

Key ExPEC and uropathogenic *E. coli*/UPEC-associated markers included genes linked to adhesion, iron acquisition, and serum resistance (e.g., *papA/C*, *sfa/focDE*, *afa/draBC*, *iutA*, *chuA*, *kpsMII*, *fyuA*, *yfcV*, *vat)* (21, 50, 51), along with additional ExPEC-associated genes (*cnf*, *clbB*, *tcpC*, *usp*, *iss*, *traT*, *neuC*, *ibeA*, *iroN*, *iucC*, *irp2*, *sitA*) to support broader pathogenicity profiling (21, 29, 51, 52).

Virulence potential was quantified using a tiered scoring system (Table S10A). Isolates carrying major DEC markers or key ExPEC/UPEC-associated virulence determinants were assigned a high virulence score (3). Isolates with secondary or supporting virulence determinants were assigned an intermediate score (2), while those with only minor virulence genes were assigned a low score (1). Isolates lacking detectable virulence determinants were assigned a score of 0. The virulence score was defined as the highest category detected per isolate, representing maximal predicted pathogenic potential. Importantly, this framework reflects genomic pathogenic potential rather than confirmed clinical virulence.

#### Antimicrobial resistance gene detection and scoring

ARGs were detected using abritamr v1.0.14 (https://github.com/MDU-PHL/abritamr) with a threshold of ≥90% sequence identity and ≥80% gene coverage. Detected genes were classified according to antimicrobial class and clinical relevance, informed by the WHO Bacterial Priority Pathogens List (53). To focus the genomic hazard assessment on clinically relevant acquired antimicrobial resistance determinants, intrinsic chromosomal resistance-associated genes commonly present in *E. coli* were excluded from ARG hazard scoring. These included genes associated with multidrug efflux systems and their regulation (*acrAB-tolC*, *emrA/B*, *mdtM*, *marA*, *soxS*, *rob,* and *evgA*), outer cell membrane permeability and cell envelope structure (*ompC*, *ompF*, *rfaG,* and *lpxM*), and intrinsic *ampC* β-lactamase of *E. coli* (*bla_EC_*) (54–57). These genes were therefore not considered acquired ARGs for hazard scoring purposes.

A hierarchical scoring system was applied to quantify ARG-associated genomic hazard based on clinical importance, mobility, and therapeutic relevance. High-hazard (score = 3) resistance determinants included carbapenemase genes (*bla*_NDM_, *bla*_KPC_, *bla*_OXA-48-like variants_), ESBLs (e.g., *bla*_CTX-M_ variants, *bla*_TEM-3/-10_, *bla*_SHV_ variants), and colistin resistance genes (*mcr-1* to *mcr-10*) (53, 58–60). These genes represent WHO critical-priority resistance determinants associated with multidrug-resistant infections and global dissemination (53, 61–63).

Moderate-hazard (score = 2) determinants included aminoglycoside resistance genes encoding 16S rRNA methyltransferases (e.g., *armA*, *rmtB*), conferring high-level aminoglycoside resistance (53, 64, 65), and fluoroquinolone resistance mediated by chromosomal quinolone resistance-determining region (QRDR) mutations. Isolates were assigned a score of 2 when they harbored ≥2 established QRDR mutations across *gyrA* and *parC*, consistent with clinically significant fluoroquinolone resistance in *E. coli*. Plasmid-mediated quinolone resistance (PMQR) genes, including *qnr* variants, *oqxAB, qepA*, and *aac*(6′)-Ib-cr, were not considered equivalent to QRDR-mediated resistance and were therefore classified separately as low-impact resistance markers (score = 1) when present. Additionally, low-hazard determinants (score = 1) included all remaining acquired ARGs not classified above. Isolates lacking detectable acquired ARGs were assigned a score of 0 (Table S10B).

To complement the ARG hazard score, a multidrug resistance (MDR) index was calculated to capture the breadth of antimicrobial resistance within each isolate. The MDR index was defined as the number of distinct antimicrobial classes for which at least one acquired resistance gene or relevant chromosomal mutation was detected per isolate. Each antimicrobial class was counted once per genome to avoid inflation due to multiple genes within the same class. The MDR index therefore represents resistance diversity rather than gene abundance.

#### Plasmid-associated hazard assessment

Plasmid-associated contigs and mobility classes (conjugative, mobilizable, or non-mobilizable) were predicted using MOB-suite v3.1.9 under default parameters (66). To capture both horizontal gene transfer potential and gene carried by the plasmid, a composite plasmid hazard score was developed. Plasmid mobility (M) was used as a proxy for horizontal gene transfer potential. Conjugative plasmids were assigned a score of 2, mobilizable plasmids a score of 1, and non-mobilizable or absent plasmids a score of 0. Gene content (G) was independently scored according to the presence and clinical relevance of ARGs and virulence factors detected on plasmid-associated contigs. Plasmids carrying no detectable ARGs or virulence factors were assigned a score of 0; plasmids carrying virulence factors only or non-clinically relevant ARGs were assigned a score of 1; plasmids carrying clinically relevant ARGs were assigned a score of 2; and plasmids carrying both clinically relevant ARGs and virulence factors were assigned a score of 3 (Table S10C). The final plasmid hazard score (*P*) was calculated as the product of mobility and gene content scores (*P* = *M* × *G*), thereby integrating both transfer potential and gene hazard into a single metric. Consequently, plasmids lacking detectable ARGs or virulence determinants received a plasmid hazard score of 0, irrespective of mobility classification, preventing highly mobile plasmids without relevant resistance or virulence cargo from being assigned elevated hazard scores. The final plasmid hazard score therefore ranged from 0 to 6, with higher scores indicating greater potential for dissemination of clinically relevant resistance and virulence determinants.

### Standardized composite genomic hazard index (exploratory metric)

To enable integrated visualization and cross-isolate comparison, a standardized composite genomic hazard index (CGHI) was derived as an exploratory metric. This index was not used as a primary endpoint but served as a relative ranking and visualization tool. Each component was first normalized to a 0–1 scale:


$$\begin{array}{ll} \text{Virulence score:} & V_{norm}=\frac{V_{score}}{V_{\max}}\\ \text{ARG hazard:} & A_{norm}=\frac{A_{score}}{A_{\max}}\\ \text{MDR index:} & M_{norm}=\frac{M_{score}}{M_{\max}}\\ \text{Plasmid hazard:} & P_{norm}=\frac{P_{score}}{P_{\max}} \end{array}$$

where *V*_max_, *A*_max_, and *P*_max_ represent the theoretical maximum possible scores for virulence (3), ARG (3), and plasmid-associated features (6), respectively. In contrast, the MDR index represents a count of resistance classes and does not have a predefined maximum within the scoring framework; *M*_max_ was therefore defined as the maximum observed MDR value within the data set (*M*_max_ = 8) and used for normalization.

The composite index was then calculated as the sum of the four normalized components using equal weighting:


$$\begin{array}{l} CGHI=V_{norm}+A_{norm}+M_{norm}+P_{norm} \end{array}$$

The CGHI ranged from 0 to 4, with higher values indicating a greater cumulative genomic hazard profile. Equal weighting was adopted as a pragmatic and transparent approach because insufficient empirical evidence exists to support differential weighting of the four genomic dimensions. Importantly, the four components were also retained as independent analytical variables to preserve interpretability. The CGHI provides a supplementary integrative representation of genomic hazard for visualization, exploratory analysis, and relative ranking rather than a definitive measure of public health risk. The framework does not account for gene expression, phenotypic resistance, infectivity, host susceptibility, exposure frequency, infective dose, environmental persistence, or disease outcome and therefore should not be interpreted as a direct measure of human health risk. Accordingly, the CGHI is best interpreted as a measure of relative genomic hazard rather than quantitative human health risk.

### Integration of genomic hazard framework with human exposure routes

To account for differences in potential human exposure, each water-use category was assigned an exposure score based on intended use and likely exposure pathways (Table S10D), following national and international guidelines for drinking, recycled, and recreational water (14, 15, 53, 67–69). Higher exposure scores indicate greater opportunities for human contact via ingestion, inhalation, or dermal exposure (70, 71). Exposure scores were converted to exposure weighting factors ranging from 0.25 to 1.0 (Table S10D). The weighting factors were applied to the composite genomic hazard index (CGHI) to generate an exposure-adjusted hazard index (EAHI):


$$\begin{array}{l} EAHI=CGHI\times E_{norm} \end{array}$$

where *E*_norm_ is the exposure weighting factor. The EAHI ranged from 0 to 4, with higher values indicating isolates that combine greater genomic hazard with greater potential for human exposure (Table S10E). The EAHI was used as a relative ranking metric to contextualize genomic hazard according to plausible human exposure pathways and was not intended as a quantitative measure of public health risk.

### Statistical analysis

All statistical analyses were conducted in R v4.4.0 (72). Differences in categorical variables were assessed using Fisher’s exact or chi-square tests (73–75). Continuous variables were analyzed using Wilcoxon rank-sum or Kruskal–Wallis tests. *E. coli* concentrations were expressed as most probable number (MPN) per 100 mL and log-transformed as log₁₀(MPN + 1) to account for the skewed distributions. As data were non-normally distributed (Shapiro–Wilk test, *P* < 0.001), non-parametric tests were used. Data visualization was performed using ggplot2 (75).

## Data Availability

Raw sequencing reads have been deposited in the NCBI Sequence Read Archive (SRA) under BioProject accession PRJNA1369737, with associated BioSample records SAMN53398571–SAMN53398754.

## SUPPLEMENTAL MATERIAL

Supplemental material aem.01342-26-s0001.docx Tables S1, S5, and S10; Fig. S1 to S4.

Table S2 aem.01342-26-s0002.xlsx Genomic characteristics and population structure of *Escherichia coli* isolates recovered from the recycled water system.

Table S3 aem.01342-26-s0003.xlsb Gene content of *Escherichia coli* genomes recovered from the recycled water system.

Table S4 aem.01342-26-s0004.xlsx ANI-based contextual genome comparisons and source attribution of *Escherichia coli* isolates recovered from the recycled water system.

Table S6 aem.01342-26-s0005.xlsx Virulence factors, antimicrobial resistance determinants, and plasmid-associated features of *Escherichia coli* isolates recovered from the recycled water system.

Table S7 aem.01342-26-s0006.xlsx Genomic hazard scoring framework for *Escherichia coli* isolates recovered from the recycled water system.

Table S8 aem.01342-26-s0007.xlsx Sample and isolate metadata for *Escherichia coli* recovered from the recycled water system.

Table S9 aem.01342-26-s0008.xlsx Contextual reference genome metadata used for comparative genomic and source attribution analyses of *Escherichia coli* isolates.

ASM does not own the copyrights to Supplemental Material that may be linked to, or accessed through, an article. The authors have granted ASM a non-exclusive, world-wide license to publish the Supplemental Material files. Please contact the corresponding author directly for reuse.
