# Supplementary material for: Whole-genome surveillance supports hazard profiling of Escherichia coli lineages in recycled water treatment systems
Source: Appl Environ Microbiol. 2026 Sep 15;92(10):e01342-26. doi: 10.1128/aem.01342-26 (PMC13688910; doi:10.1128/aem.01342-26)
Supplement: Supplementary file 1 [file aem.01342-26-s0001.docx]

**Supplementary material**

**Table S1A.** Summary statistics of *E. coli* loads (MPN/100mL) by sample collection sites. Values represent log-transformed (log₁₀[MPN + 1]) and raw MPN (Most Probable Number) measurements for each source (W1, W2, W3). For each site, the number of samples (n), mean, median, interquartile range (Q1–Q3), and standard deviation (SD) are reported.

| **Sample site** | **Sample number** | **Log_10_(MPN+1)** | | | | | **MPN** | |
| --- | --- | --- | --- | --- | --- | --- | --- | --- |
|  |  | **Mean** | **Median** | **Q1** | **Q3** | **SD** | **Mean** | **Median** |
| W1 | 36 | 1.799137 | 1.816241 | 1.565247 | 2.065384 | 0.366588 | 86.16286 | 64.5 |
| W2 | 32 | 0.094087 | 0 | 0 | 0.176091 | 0.219164 | 0.578125 | 0 |
| W3 | 84 | 0.435449 | 0.30103 | 0.176091 | 0.63648 | 0.422843 | 5.088095 | 1 |

**Figure S1A.** Boxplot showing the log-transformed *E. coli* counts log₁₀(MPN + 1) by water source (W1, W2, W3). Points represent individual samples, and boxes indicate the interquartile range with medians. Pairwise comparisons were conducted using the Mann–Whitney U test due to non-normal distribution in some groups. Asterisks (*) denote statistically significant differences (p < 0.05).

**Table S1B.** Summary statistics of log-transformed *E. coli* concentrations (MPN/100mL) by season, including sample size (n), mean, median, interquartile range (Q1–Q3), and standard deviation (SD).

| **Season** | **Sample number** | **Mean** | **Median** | **Q1** | **Q3** | **SD** |
| --- | --- | --- | --- | --- | --- | --- |
| Spring | 16 | 0.683989 | 0.349485 | 0 | 1.345053 | 0.741487 |
| Summer | 56 | 0.731982 | 0.477121 | 0.30103 | 0.866257 | 0.616031 |
| Autumn | 33 | 0.851871 | 0.477121 | 0.30103 | 1.579784 | 0.761564 |
| Winter | 14 | 1.392923 | 1.906256 | 0.345053 | 2.140311 | 0.974628 |

**Table S1C. MPN by season.** Post-hoc Dunn’s test results for pairwise comparisons of *E. coli* loads (MPN/100mL) between seasons following a Kruskal–Wallis test. Reported are unadjusted and Bonferroni-adjusted p-values. No comparisons remained statistically significant after correction.

| **Comparison** | **Z** | **p (unadj)** | **p (adj)** |
| --- | --- | --- | --- |
| Autumn - Spring | 0.996641 | 0.318939 | 1 |
| Autumn - Summer | 0.035286 | 0.971852 | 1 |
| Spring - Summer | -1.04373 | 0.296611 | 1 |
| Autumn - Winter | -1.83424 | 0.066618 | 0.399709 |
| Spring - Winter | -2.42826 | 0.015172 | 0.091029 |
| Summer - Winter | -1.98383 | 0.047275 | 0.28365 |

**Figure S1B.** Seasonal variation in *E. coli* counts (log₁₀[MPN + 1]) at sources W1 and W3. Boxplots display the distribution of microbial concentrations across four seasons. Points represent individual samples with added jitter for clarity. A Kruskal–Wallis test was used to assess overall differences across seasons (p = 0.102).

**Figure S1C.** Correlation between pH and *E. coli* counts (log₁₀[MPN + 1]) in water samples from sources W1 and W3. Scatter plots with fitted Spearman correlation regression lines and 95% confidence intervals are shown for each source (colors and shapes differentiate sites). Across all samples, a significant positive correlation was observed between pH and microbial counts (ρ = 0.413, p < 0.001).

**Figure S1D.** Correlation between temperature and *E. coli* counts (log₁₀[MPN + 1]) in water samples from sources W1 and W3 with regression lines and 95% confidence intervals for W1 and W3. A weak negative correlation was observed overall (R = –0.26, p = 0.040).

**Figure S1E.** Scatter plot showing the correlation between dissolved oxygen (DO, mg/L) and *E. coli* counts (log₁₀[MPN + 1]) in water samples from sources W1 and W3. Points are coloured and shaped by source, with fitted Spearman correlation regression lines and 95% confidence intervals. No significant correlation was found between DO and microbial counts across all samples (ρ = –0.097, p = 0.456).

**Figure S2**. **Gene richness of** E. coli **isolates.** Boxplots represent the distribution of gene counts for each collection site (W1, W2, W3), with boxes showing the interquartile range and the horizontal line indicating the median. Individual data points are overlaid as grey dots to show the spread and variability within each group. Colours correspond to the sample collection site as indicated in the plot.

**Figure S3A.** Source attribution of *E. coli* isolates. Attribution was assigned based on the highest average nucleotide identity (ANI) to reference genomes. Most isolates matched companion-animal strains (37.8%), followed by wild birds (24.4%), humans (13.9%), porcine (12.8%), cattle (3.3%), poultry (6.7%), wastewater (0.6%), and feline sources (0.6%).

|  | Count |
| --- | --- |
| Comp_Feline | 1 |
| Wastewater | 1 |
| Cattle | 6 |
| Poulty | 12 |
| Porcine | 23 |
| Human | 25 |
| Wild_Avian | 44 |
| Comp_Canine | 68 |

**Figure S3B.** **Source attribution profiles of Escherichia coli isolates at sites W1 and W3.**
Stacked bar plots show the proportional distribution of predicted potential sources for E. coli isolates recovered from W1 and W3. Bar heights represent the relative proportion of isolates attributed to each source category within each site, scaled to 100%.

**Table S5A.** P**airwise SNP distances among ST162 isolates across collection sites** (W1, W3) and sampling dates. Columns list the isolate identifiers (Isolate A and B), collection site and date, phylogroup, pairwise SNP distance, and type of comparison (Intra-site: isolates from the same site; Inter-site: isolates from different sites). SNP distances reflect the genomic divergence between isolate pairs, with lower values indicating closer relatedness and potential recent transmission or persistence.

| **Isolate A** | **Collection site A** | **Collection date A** | **Phylogroup A** | **Isolate B** | **Collection site B** | **Collection date B** | **Phylogroup B** | **SNP Distance** | **Type** |
| --- | --- | --- | --- | --- | --- | --- | --- | --- | --- |
| 14241049P0 | W2 | 8/01/2025 | B1 | 14213319P0 | W3 | 31/12/2024 | B1 | 0 | Inter-site |
| 1908834028 | W1 | 15/05/2024 | B1 | 14242707P0 | W3 | 12/12/2024 | B1 | 1 | Inter-site |
| 1908834028 | W1 | 15/05/2024 | B1 | 14370875P0 | W3 | 2/12/2024 | B1 | 9 | Inter-site |
| 1908834028 | W1 | 15/05/2024 | B1 | 14242374P0 | W3 | 2/12/2024 | B1 | 11 | Inter-site |
| 1908834028 | W1 | 15/05/2024 | B1 | 14213319P0 | W3 | 31/12/2024 | B1 | 20 | Inter-site |
| 1908834028 | W1 | 15/05/2024 | B1 | 14241049P0 | W2 | 8/01/2025 | B1 | 20 | Inter-site |
| 14370875P0 | W3 | 2/12/2024 | B1 | 14241049P0 | W2 | 8/01/2025 | B1 | 21 | Inter-site |
| 14242707P0 | W3 | 12/12/2024 | B1 | 14241049P0 | W2 | 8/01/2025 | B1 | 21 | Inter-site |
| 14242374P0 | W3 | 2/12/2024 | B1 | 14241049P0 | W2 | 8/01/2025 | B1 | 23 | Inter-site |
| 14369008P0 | W1 | 30/05/2025 | B1 | 14216183P0 | W3 | 31/12/2024 | B1 | 188 | Inter-site |
| 1908834028 | W1 | 15/05/2024 | B1 | 14216183P0 | W3 | 31/12/2024 | B1 | 3700 | Inter-site |
| 14370875P0 | W3 | 2/12/2024 | B1 | 14369008P0 | W1 | 30/05/2025 | B1 | 3702 | Inter-site |
| 14241049P0 | W2 | 8/01/2025 | B1 | 14216183P0 | W3 | 31/12/2024 | B1 | 3706 | Inter-site |
| 14369008P0 | W1 | 30/05/2025 | B1 | 14242707P0 | W3 | 12/12/2024 | B1 | 3709 | Inter-site |
| 14369008P0 | W1 | 30/05/2025 | B1 | 14242374P0 | W3 | 2/12/2024 | B1 | 3712 | Inter-site |
| 14369008P0 | W1 | 30/05/2025 | B1 | 14241049P0 | W2 | 8/01/2025 | B1 | 3715 | Inter-site |
| 14369008P0 | W1 | 30/05/2025 | B1 | 14213319P0 | W3 | 31/12/2024 | B1 | 3715 | Inter-site |
| 14370875P0 | W3 | 2/12/2024 | B1 | 14242374P0 | W3 | 2/12/2024 | B1 | 2 | Intra-site |
| 14370875P0 | W3 | 2/12/2024 | B1 | 14242707P0 | W3 | 12/12/2024 | B1 | 10 | Intra-site |
| 14242707P0 | W3 | 12/12/2024 | B1 | 14242374P0 | W3 | 2/12/2024 | B1 | 12 | Intra-site |
| 14242707P0 | W3 | 12/12/2024 | B1 | 14213319P0 | W3 | 31/12/2024 | B1 | 21 | Intra-site |
| 14370875P0 | W3 | 2/12/2024 | B1 | 14213319P0 | W3 | 31/12/2024 | B1 | 21 | Intra-site |
| 14242374P0 | W3 | 2/12/2024 | B1 | 14213319P0 | W3 | 31/12/2024 | B1 | 23 | Intra-site |
| 14370875P0 | W3 | 2/12/2024 | B1 | 14216183P0 | W3 | 31/12/2024 | B1 | 3693 | Intra-site |
| 14242707P0 | W3 | 12/12/2024 | B1 | 14216183P0 | W3 | 31/12/2024 | B1 | 3700 | Intra-site |
| 14242374P0 | W3 | 2/12/2024 | B1 | 14216183P0 | W3 | 31/12/2024 | B1 | 3703 | Intra-site |
| 14216183P0 | W3 | 31/12/2024 | B1 | 14213319P0 | W3 | 31/12/2024 | B1 | 3706 | Intra-site |
| 1908834028 | W1 | 15/05/2024 | B1 | 14369008P0 | W1 | 30/05/2025 | B1 | 3709 | Intra-site |

**Table S5B.** P**airwise SNP distances among ST155 isolates across collection sites** (W1, W3) and sampling dates. Columns list the isolate identifiers (Isolate A and B), collection site and date, phylogroup, pairwise SNP distance, and type of comparison (Intra-site: isolates from the same site; Inter-site: isolates from different sites). SNP distances reflect the genomic divergence between isolate pairs, with lower values indicating closer relatedness and potential recent transmission or persistence.

| **Isolate A** | **Collection site A** | **Collection site A** | **Phylogroup A** | **Isolate B** | **Collection site B** | **Collection site B** | **Phylogroup B** | **SNP Distance** | **Type** |
| --- | --- | --- | --- | --- | --- | --- | --- | --- | --- |
| 14250896P0 | W1 | 14/05/2025 | B1 | 14369846P0 | W1 | 14/05/2025 | B1 | 1 | Intra-site |
| 14241101P0 | W3 | 17/01/2025 | B1 | 14371386P0 | W3 | 17/01/2025 | B1 | 3 | Intra-site |
| 14240058P0 | W3 | 8/01/2025 | B1 | 14370986P0 | W3 | 23/12/2024 | B1 | 141 | Intra-site |
| 14213301P0 | W3 | 31/01/2025 | B1 | 14370986P0 | W3 | 23/12/2024 | B1 | 203 | Intra-site |
| 14213301P0 | W3 | 31/01/2025 | B1 | 14240058P0 | W3 | 8/01/2025 | B1 | 210 | Intra-site |
| 14250896P0 | W1 | 14/05/2025 | B1 | 14369076P0 | W1 | 4/12/2024 | B1 | 867 | Intra-site |
| 14369076P0 | W1 | 4/12/2024 | B1 | 14369846P0 | W1 | 14/05/2025 | B1 | 867 | Intra-site |
| 14213301P0 | W3 | 31/01/2025 | B1 | 14371386P0 | W3 | 17/01/2025 | B1 | 1064 | Intra-site |
| 14213301P0 | W3 | 31/01/2025 | B1 | 14241101P0 | W3 | 17/01/2025 | B1 | 1071 | Intra-site |
| 14370986P0 | W3 | 23/12/2024 | B1 | 14371386P0 | W3 | 17/01/2025 | B1 | 1136 | Intra-site |
| 14240058P0 | W3 | 8/01/2025 | B1 | 14371386P0 | W3 | 17/01/2025 | B1 | 1141 | Intra-site |
| 14241101P0 | W3 | 17/01/2025 | B1 | 14370986P0 | W3 | 23/12/2024 | B1 | 1142 | Intra-site |
| 14240058P0 | W3 | 8/01/2025 | B1 | 14241101P0 | W3 | 17/01/2025 | B1 | 1147 | Intra-site |
| 14369846P0 | W1 | 14/05/2025 | B1 | 14371386P0 | W3 | 17/01/2025 | B1 | 1599 | Inter-site |
| 14250896P0 | W1 | 14/05/2025 | B1 | 14371386P0 | W3 | 17/01/2025 | B1 | 1600 | Inter-site |
| 14241101P0 | W3 | 17/01/2025 | B1 | 14369846P0 | W1 | 14/05/2025 | B1 | 1606 | Inter-site |
| 14241101P0 | W3 | 17/01/2025 | B1 | 14250896P0 | W1 | 14/05/2025 | B1 | 1607 | Inter-site |
| 14213301P0 | W3 | 31/01/2025 | B1 | 14369846P0 | W1 | 14/05/2025 | B1 | 1686 | Inter-site |
| 14213301P0 | W3 | 31/01/2025 | B1 | 14250896P0 | W1 | 14/05/2025 | B1 | 1687 | Inter-site |
| 14213301P0 | W3 | 31/01/2025 | B1 | 14369076P0 | W1 | 4/12/2024 | B1 | 1712 | Inter-site |
| 14369846P0 | W1 | 14/05/2025 | B1 | 14370986P0 | W3 | 23/12/2024 | B1 | 1758 | Inter-site |
| 14250896P0 | W1 | 14/05/2025 | B1 | 14370986P0 | W3 | 23/12/2024 | B1 | 1759 | Inter-site |
| 14240058P0 | W3 | 8/01/2025 | B1 | 14369846P0 | W1 | 14/05/2025 | B1 | 1774 | Inter-site |
| 14240058P0 | W3 | 8/01/2025 | B1 | 14250896P0 | W1 | 14/05/2025 | B1 | 1775 | Inter-site |
| 14369076P0 | W1 | 4/12/2024 | B1 | 14370986P0 | W3 | 23/12/2024 | B1 | 1786 | Inter-site |
| 14240058P0 | W3 | 8/01/2025 | B1 | 14369076P0 | W1 | 4/12/2024 | B1 | 1800 | Inter-site |
| 14369076P0 | W1 | 4/12/2024 | B1 | 14371386P0 | W3 | 17/01/2025 | B1 | 1978 | Inter-site |
| 14241101P0 | W3 | 17/01/2025 | B1 | 14369076P0 | W1 | 4/12/2024 | B1 | 1984 | Inter-site |

**Figure S4.** Prevalence of the top 10 antibiotic subclasses in E. coli isolates across collection sites (W1, W2, W3). Bars represent the percentage of isolates carrying at least one ARG assigned to each antibiotic subclass at each site. The colistin subclass was predominantly represented by chromosomal pmrB variants rather than acquired mcr-type colistin resistance genes.

**Table S10A.** Classification of *E. coli* virulence factors (VFs) by encoded function, pathotype association, and hazard category.

| **Gene(s)** | **Encoded Function** | **Hazard score** | **Notes** | **Supporting references** |
| --- | --- | --- | --- | --- |
| *eae* | Intimin (outer membrane adhesin) | 3 | Essential for epithelial attachment | 1 – 4 |
| *ehxA* | Enterohemolysin |  | Epithelial damage, systemic effects |  |
| *bfpA* | Bundle-forming pilin |  | Critical for localized adherence |  |
| *stx1, stx2* | Shiga toxins |  | Central to HUS and systemic toxicity |  |
| *aggR* | Global regulator |  | Activates virulence genes *aaiC*, *aatA* |  |
| *aaiC, aatA* | Secretion/adherence proteins |  | AggR-regulated effectors |  |
| *afa, daaE* | A fimbrial adhesins |  | Linked to persistent pediatric diarrhea |  |
| *eltA/B, estA/B* | Heat-labile/stable enterotoxins |  | Cause secretory diarrhea |  |
| *ipaH, virF, itcA* | Invasion associated genes |  | Mediate epithelial cell invasion |  |
| *papA/C* | P fimbriae (adhesins) |  | Urinary tract colonization, biofilm formation | 2, 4 – 10 |
| *sfa/focDE* | S and F1C fimbriae |  | CNS and uroepithelial adhesion |  |
| *afa/draBC* | Dr-binding adhesins |  | Associated with invasive infections |  |
| *kpsMII* | Group 2 capsular polysaccharide synthesis |  | Serum resistance, immune evasion |  |
| *fyuA* | Yersiniabactin siderophore system |  | associated with iron uptake |  |
| *chuA* | Outer membrane hemin receptor |  | outer membrane protein |  |
| *vat* | Vacuolating autotransporter toxin |  | Tissue damage, immune modulation |  |
| *hlyE* | Hemolysin/cytolysin |  | Lysing red blood cells and macrophages |  |
| *yfcV* | Fimbrial operon |  | adhesion to surfaces and host cells |  |
| *tir* | Translocated intimin receptor | 2 | Works with *eae* for intimate adhesion | 1, 2, 6, 9 |
| *cfaI/II* | Colonization factor antigens |  | Critical for adherence to intestinal mucosa |  |
| *iutA* | Iron uptake systems |  | Enhance survival in host environments |  |
| *espA/B/D* | Type III secretion system components |  | Facilitate effector delivery |  |
| *espP, saa, nle* | Additional toxins and effectors |  | Support STEC virulence |  |
| *ial, ipaB/C/D* | Invasion-related proteins |  | Mediate epithelial cell invasion |  |
| *aggA/B* | Aggregative adherence fimbriae |  | Promote intestinal colonization |  |
| *aap* | Dispersin (antiaggregation protein) |  | Facilitates biofilm dispersal |  |
| *sat* | Secreted autotransporter toxin |  | Causes epithelial damage |  |
| *fimH, focG, hra, ibeA, iha, ireA, iroN, irp2, iss, iucC, kpsE, lpfA, mcbA, mchB, mcmA, neuC, ompT, pic, sitA, senB, tcpC, terC, traT, usp, astA, cba, cea, cia, cib, clbB, cnf, cvaC, espI, etsC* | Adhesins, toxins, iron acquisition, serum resistance, etc. | 1 |  | 4 - 6, 8, 9 |

**Table S10B.** Classification of antibiotic resistance genes (ARGs) within assessment framework. *E. coli* isolates carrying any ARGs not listed in this table were assigned a score of 1 (low hazard), whereas isolates with no detected ARGs (ND) were assigned a score of 0

| **Gene(s)** | **Encoded Resistance** | **Hazard Classification** | **Hazard score** | **Notes** | **Supporting References** |
| --- | --- | --- | --- | --- | --- |
| *bla*_NDM_ | Carbapenem resistance | High | 3 | Carbapenemases; plasmid-mediated global spread; major drivers of carbapenem resistance, WHO critical pathogen.  KPC carbapenemase; major hospital-associated resistance determinant.  OXA-48-like carbapenemases; global dissemination. | 11 – 14 |
| *bla*_KPC_ |  |  |  |  |  |
| *bla*_VIM_ |  |  |  |  |  |
| *bla*_IMP_ |  |  |  |  |  |
| *bla*_OXA-48_*-*like (e.g., OXA-48, OXA-181, OXA-244) |  |  |  |  |  |
| *bla*_CTX-M_ variants (e.g., CTX-M-15, -14, -27) | Extended-spectrum β-lactamases (ESBL) |  |  | Major ESBL family; global dissemination; associated with MDR lineages (e.g., ST131) | 13, 15 – 18 |
| *bla*_SHV_ *varians* (e.g., SHV-12, -2, -5) |  |  |  | ESBL-associated SHV variants; plasmid-mediated third-generation cephalosporin resistance. |  |
| *bla*_TEM_ variant (e.g., TEM-3, -10, -52) |  |  |  | ESBL-associated TEM derivatives with extended-spectrum β-lactamase activity. |  |
| *mcr-1 - mcr-10* | Colistin (polymyxin) resistance |  |  | Last-resort antimicrobial resistance; plasmid-mediated colistin resistance. | 19 - 21 |
| *armA, rmtB* | Aminoglycoside resistance | Moderate | 2 | 16S rRNA methyltransferases conferring high-level aminoglycoside resistance. | 13, 22, 23 |
| *gyrA+parC QRDR mutations* | Fluoroquinolone resistance |  |  | Assigned when ≥2 established QRDR mutations (gyrA/parC) are present; associated with clinically significant fluoroquinolone resistance in E. coli |  |
| PMQR genes (qnr variants, oqxAB, qepA, aac(6')-Ib-cr) | Fluoroquinolone reduced susceptibility | Low | 1 | Plasmid-mediated quinolone resistance; not equivalent to QRDR-mediated resistance | 22 – 24 |
| Other acquired ARGs not classified above | Various |  |  | Includes remaining acquired resistance genes not meeting high or moderate hazard criteria |  |
| No detectable acquired ARGs | None | None | 0 | No acquired ARGs detected after filtering intrinsic genes |  |

**Note:** In addition to the hazard score in table S10B (Layer 1), a multidrug resistance (MDR) index was calculated per isolate based on the number of antimicrobial classes represented by detected resistance determinants (Layer 2)

**Table S10C.** Plasmid-associated hazard scoring based on plasmid mobility and carriage of antimicrobial resistance and virulence genes.

| **Component** | **Definition** | **Biological interpretation** | **Score** |
| --- | --- | --- | --- |
| **Plasmid status / mobility (M)** | Conjugative plasmid | Self-transmissible plasmid | 2 |
|  | Mobilizable plasmid | Can be mobilized in trans | 1 |
|  | No plasmid detected | No plasmid-mediated HGT potential | 0 |
|  | Non-mobilizable plasmid | Present but no transfer potential |  |
| **Gene content (G)** | Clinically relevant ARGs **and** clinically relevant virulence genes | Combined resistance and pathogenicity potential | 3 |
|  | Clinically relevant ARGs **or** clinically relevant virulence genes | Resistance or pathogenicity potential | 2 |
|  | Intrinsic ARGs and/or minor virulence factors only | Baseline biological function, low clinical relevance | 1 |
|  | No virulence gene or ARG detected | No hazard-associated cargo | 0 |

**Table S10D**. Exposure scoring framework applied across water usage categories. The influent (W1) was classified as Class C recycled water, while the final effluent at site W3 represented Class A recycled water. Samples collected post-UV treatment (W2a), and post UV and chlorine injection (W2b) could be classified as Class B recycled water.

| **Water type/ usage** | **Description** | **Exposure characteristics** | **Exposure score** | ***Exposure adjustment factor (E_norm_)** |
| --- | --- | --- | --- | --- |
| Drinking water | Treated potable water intended for direct human consumption | Continuous ingestion; high-frequency, direct exposure | 4 | 1.00 |
| Recycled water (Class A) | Irrigation of edible produce eaten raw; residential reuse (toilet flushing, garden watering, laundry); urban irrigation (parks, golf courses) | Frequent dermal, potential ingestion, and aerosol exposure via direct or indirect contact | 3 | 0.75 |
| Recycled water (Class B) | Irrigation of crops cooked before consumption; dairy pastures | Indirect ingestion; moderate occupational exposure | 2 | 0.50 |
| Recreational water (primary contact) | Swimming, water sports, direct contact with surface waters | Episodic but direct exposure via ingestion and dermal contact | 2.5 | 0.625 |
| Recreational water (secondary contact) | Boating, fishing, incidental contact only | Limited or infrequent exposure | 2 | 0.50 |
| Recycled water (Class C) | Irrigation of fodder, cereals, non-food crops, turf farms (controlled access) | Limited occupational exposure; minimal public contact | 1.5 | 0.375 |
| Recycled water (Class D) | Industrial or non-food uses (dust suppression, construction water) | Negligible contact or ingestion pathways | 1 | 0.250 |
| Raw sewage | Raw influent, sludge, or pre-treatment effluent | Excluded from end-use matrix due to baseline raw pathogen loads | - | - |

* Exposure scores (1–4) were normalised by dividing by the maximum exposure score (4), producing a **dimensionless exposure weight** between 0 and 1

**Table 10E.** Hazard classification scheme for the Exposure-Adjusted Hazard Index (EAHI). Categories were used to rank environmental E. coli isolates according to their combined genomic hazard profile (virulence, antimicrobial resistance, multidrug resistance, and plasmid-associated transfer potential) and estimated human exposure potential. Classifications represent relative hazard rankings and do not constitute direct measures of public health risk.

| **Final score range** | **Hazard classification** | **Public health & Operational meaning** |
| --- | --- | --- |
| 0.00 – 1.00 | Low Hazard | Commensal/mild E. coli strains OR hazardous strains well-contained in low-exposure settings (e.g., Class D industrial use) |
| 1.01 – 2.00 | Moderate Hazard | Strains possessing moderate virulence/resistance in public areas (Class A/B), OR high-hazard strains limited to restricted agricultural sites |
| 2.01 – 3.00 | High Hazard | Potentially pathogenic or highly resistant strains entering high-exposure pathways (Class A or Recreational waters) |
| 3.01 – 4.00 | Critical Hazard | Highly virulent, multi-drug resistant strains carrying mobile plasmids detected in high-frequency exposure pathways (Drinking water or Class A) |

**References**

1. Kaper JB, Nataro JP, Mobley HL. (2004). Pathogenic *Escherichia coli*. *Nat Rev Microbiol*, *2*(2), 123-140.
2. Pakbin B, Bruck WM, Rossen JWA. (2021). Virulence Factors of Enteric Pathogenic *Escherichia coli*: A RevieW*Int J Mol Sci*, *22*(18).
3. Kaper JB, & Karmali MA. (2008). The continuing evolution of a bacterial pathogen. *Proc Natl Acad Sci U S A*, *105*(12), 4535-4536.
4. Vanstokstraeten R, Crombe F, Pierard D, Moral C, Wybo I, De Geyter D, Janssen T, Caljon B, Demuyser T. (2022). Molecular characterization of extraintestinal and diarrheagenic Escherichia coli blood isolates. *Virulence*, *13*(1), 2032-2041.
5. Johnson JR, Johnston BD, Porter S, Thuras P, Aziz M, Price LB. (2019). Accessory Traits and Phylogenetic Background Predict *Escherichia coli* Extraintestinal Virulence better than does Ecological Source. *J Infect Dis*, *219*(1), 121-132.
6. Lagerstrom KM, Scales NC, Hadly EA. (2024). Impressive pan-genomic diversity of *E. coli* from a wild animal community near urban development reflects human impacts. *iScience*, *27*(3), 109072.
7. Mageiros L, Meric G, Bayliss SC, Pensar J, Pascoe B, Mourkas E, Calland JK, Yahara K, Murray S, Wilkinson TS, Williams LK, Hitchings MD, Porter J, Kemmett K, Feil EJ, Jolley KA, Williams NJ, Corander J, Sheppard SK. (2021). Genome evolution and the emergence of pathogenicity in avian *Escherichia coli*. *Nat Commun*, *12*(1), 765.
8. Tetzschner AMM, Johnson JR, Johnston BD, Lund O, Scheutz F. (2020). In Silico Genotyping of Escherichia coli Isolates for Extraintestinal Virulence Genes by Use of Whole-Genome Sequencing Data. *J Clin Microbiol*, *58*(10).
9. Sora VM, Meroni G, Martino PA, Soggiu A, Bonizzi L, Zecconi A. (2021). Extraintestinal Pathogenic *Escherichia coli*: Virulence Factors and Antibiotic Resistance. *Pathogens*, *10*(11).
10. Spurbeck RR, Dinh Jr PC, Walk ST, Stapleton AE, Hooton TM, Nolan LK, Kim KS, Johnson JR, & Mobley HL. (2012). Escherichia coli isolates that carry vat, fyuA, chuA, and yfcV efficiently colonize the urinary tract. *Infect Immun*, *80*(12), 4115-4122
11. Logan LK, Weinstein RA. (2017). The Epidemiology of Carbapenem-Resistant Enterobacteriaceae: The Impact and Evolution of a Global Menace. *J Infect Dis*, *215*(suppl_1), S28-S36.
12. Nordmann P, Naas T, Poirel L. (2011). Global spread of Carbapenemase-producing Enterobacteriaceae. *Emerg Infect Dis*, *17*(10), 1791-1798.
13. World Health Organization. (2024). *Guidelines for drinking-water quality: Small water supplies*. Switzerland: World Health Organization Retrieved from <https://www.who.int/publications/i/item/9789240088740>
14. Wise MG, Karlowsky JA, Mohamed N, Hermsen ED, Kamat S, Townsend A, Brink A, Soriano A, Paterson DL, Moore LSP, & Sahm DF. (2024). Global trends in carbapenem- and difficult-to-treat-resistance among World Health Organization priority bacterial pathogens: ATLAS surveillance program 2018-2022. *J Glob Antimicrob Resist*, *37*, 168-175
15. Bevan ER, Jones AM, & Hawkey PM. (2017). Global epidemiology of CTX-M beta-lactamases: temporal and geographical shifts in genotype. *J Antimicrob Chemother*, *72*(8), 2145-2155.
16. Canton R, & Coque TM. (2006). The CTX-M beta-lactamase pandemic. *Curr Opin Microbiol*, *9*(5), 466-475.
17. Ramatla T, Tawana M, Lekota KE, & Thekisoe O. (2023). Antimicrobial resistance genes of Escherichia coli, a bacterium of "One Health" importance in South Africa: Systematic review and meta-analysis. *AIMS Microbiol*, *9*(1), 75-89.
18. Zhang AN, Gaston JM, Dai CL, Zhao S, Poyet M, Groussin M, Yin X, Li LG, van Loosdrecht MCM, Topp E, Gillings MR, Hanage WP, Tiedje JM, Moniz K, Alm EJ, & Zhang T. (2021). An omics-based framework for assessing the health risk of antimicrobial resistance genes. *Nat Commun*, *12*(1), 4765.
19. Liu JH, Liu YY, Shen YB, Yang J, Walsh TR, Wang Y, Shen J. (2024). Plasmid-mediated colistin-resistance genes: mcr. *Trends Microbiol*, *32*(4), 365-378.
20. Liu YY, Wang Y, Walsh TR, Yi LX, Zhang R, Spence J, Doi Y, Tian G, Dong B, Huang X, Yu LF, Gu D, Ren H, Chen X, Lv L, He D, Zhou H, Liang Z, Liu, JH, Shen J. (2016). Emergence of plasmid-mediated colistin resistance mechanism MCR-1 in animals and human beings in China: a microbiological and molecular biological study. *Lancet Infect Dis*, *16*(2), 161-168.
21. Shen Y, Zhou H, Xu J, Wang Y, Zhang Q, Walsh TR, Shao B, Wu C, Hu Y, Yang L, Shen Z, Wu Z, Sun Q, Ou Y, Wang Y, Wang S, Wu Y, Cai C, Li J, Wang Y. (2018). Anthropogenic and environmental factors associated with high incidence of mcr-1 carriage in humans across China. *Nat Microbiol*, *3*(9), 1054-1062.
22. Doma AO, Popescu R, Mituletu M, Muntean D, Degi J, Boldea MV, Radulov I, Dumitrescu E, Muselin F, Puvaca N, Cristina RT. (2020). Comparative Evaluation of qnrA, qnrB, and qnrS Genes in Enterobacteriaceae Ciprofloxacin-Resistant Cases, in Swine Units and a Hospital from Western RomaniA *Antibiotics (Basel)*, *9*(10).
23. Kherroubi L, Bacon J, Rahman KM. (2024). Navigating fluoroquinolone resistance in Gram-negative bacteria: a comprehensive evaluatioN*JAC Antimicrob Resist*, *6*(4), dlae127.
24. Tarek MH, Garner E. (2023). A proposed framework for the identification of indicator genes for monitoring antibiotic resistance in wastewater: Insights from metagenomic sequencing. *Sci Total Environ*, *854*, 158698.
